# Molecular Basis and Mechanistic Insights into *Ascophyllum nodosum* Extract-Mediated Regulation of Plant Growth, Nutrient Acquisition, and Stress Responses

**DOI:** 10.3390/plants15121913

**Published:** 2026-06-20

**Authors:** Prabhaharan Renganathan, Lira A. Gaysina, Juan Carlos Sainz-Hernández, Edgar Omar Rueda Puente

**Affiliations:** 1 Department of Bioecology and Biological Education, M. Akmullah Bashkir State Pedagogical University, 450000 Ufa, Russia; prabhaharan06@gmail.com (P.R.); lira.gaisina@gmail.com (L.A.G.); 2 All-Russian Research Institute of Phytopathology, 143050 Bolshye Vyazemy, Russia; 3 Phystech School of Biological and Medical Physics, Moscow Institute of Physics and Technology, 141701 Dolgoprudny, Russia; 4 Centro Interdisciplinario de Investigación para el Desarrollo Integral Regional Unidad Sinaloa, Instituto Politécnico Nacional, Guasave 81049, Mexico; 5 Departamento de Agricultura y Ganadería, Universidad de Sonora, Hermosillo 83000, Mexico

**Keywords:** *Ascophyllum nodosum* extract, biostimulants, plant signaling, transcriptional regulation, nutrient acquisition, stress tolerance, multi-omics, molecular mechanisms

## Abstract

*Ascophyllum nodosum* extracts (ANE) are widely used biostimulants associated with improvements in plant growth, productivity, nutrient acquisition, and abiotic stress tolerance. However, the molecular mechanisms linking extract composition to plant signaling and physiological responses remain incompletely resolved. ANE contains a complex mixture of bioactive constituents, including polysaccharides, osmolytes, phenolic compounds, and phytohormone-like molecules. Their composition varies according to biomass source, environmental conditions, and extraction methodology, contributing to variability in biological activity. Current evidence suggests that ANE functions mainly as a signaling modulator rather than a direct nutrient source. ANE treatment has been associated with early cellular responses, including cytosolic Ca^2+^ influx, reactive oxygen species (ROS) generation, and mitogen-activated protein kinase (MAPK)-associated signaling events. However, many proposed mechanisms remain unresolved, and a considerable proportion of the available mechanistic evidence originates from studies using purified ANE-derived polysaccharides or related elicitor systems. ANE-associated responses include modulation of nutrient transport, primary metabolism, hormonal regulation, transcriptional reprogramming, and stress-responsive pathways, contributing to improved root development, nutrient acquisition, and defense-related responses. Nevertheless, limited knowledge of receptor-mediated perception mechanisms, signaling hierarchies, and extract-dependent variability continues to constrain mechanistic understanding and reproducibility. Future research should prioritize receptor identification, bioassay-guided fractionation, integrated multi-omics approaches, and improved standardization of extraction and formulation procedures. These advances will be essential for establishing robust mechanistic models and supporting the development of evidence-based ANE biostimulants for sustainable crop production.

## 1. Introduction

The growing demand for sustainable agriculture, together with environmental and economic constraints associated with synthetic fertilizers and agrochemicals [1], has intensified interest in alternative approaches to improve crop productivity. Soil degradation, nutrient leaching, and eutrophication continue to threaten agricultural sustainability [2]. Consequently, attention has been directed toward environmentally friendly strategies that improve plant performance under abiotic stresses, including drought, salinity, heat, and oxidative stress [3,4]. In this context, biostimulants have emerged as important agricultural inputs that improve plant growth, nutrient-use efficiency, and stress tolerance through physiological and biochemical processes distinct from conventional fertilization [5,6]. Rather than functioning primarily as nutrient sources, biostimulants influence signal transduction, gene expression, and metabolic pathways involved in photosynthesis, phytohormonal regulation, and reactive oxygen species (ROS) homeostasis [7,8].

Among the various biostimulant categories, seaweed-derived formulations, particularly those obtained from the brown macroalga *Ascophyllum nodosum*, have received considerable attention because of their effects on plant growth and stress tolerance [9]. *A. nodosum* is widely distributed along North Atlantic coastlines and is characterized by high biomass productivity and tolerance to environmental stress [10,11,12]. Its biochemical composition includes polysaccharides (alginates, laminarin, and fucoidan), osmolytes such as mannitol, phenolic compounds including phlorotannins, and low concentrations of phytohormone-like molecules [13,14,15]. These compounds are associated with osmotic adjustment, redox homeostasis, and stress-related signaling pathways [16,17,18]. However, the composition of *A. nodosum* extracts (ANE) varies with extraction methods, seasonal fluctuations, and environmental conditions, resulting in differences in metabolite abundance and biological activity [19]. In addition, mechanistic evidence has been generated using both whole ANE formulations and purified constituent fractions, which complicates direct interpretation of ANE-mediated responses.

Several studies have shown that ANE promotes plant growth in diverse crop species by enhancing root development, nutrient uptake, photosynthetic activity, and reproductive performance [20,21,22]. ANE also improves tolerance to drought, salinity, and nutrient deficiency through its effects on antioxidant metabolism, physiological stability, and plant–microbe interactions in the rhizosphere [23,24,25]. However, the molecular basis of ANE-mediated responses remains unclear. Most studies have focused on individual physiological traits or signaling events without integrating molecular, physiological, and agronomic responses into a unified framework [26,27].

Recent molecular and omics-based studies have indicated that ANE is associated with phytohormonal regulation, ROS signaling, and transcriptional control of genes involved in plant growth and stress responses [28,29]. However, the mechanisms linking ANE perception with downstream regulatory networks remain poorly understood [27]. In particular, the receptors involved in the perception of polysaccharide elicitors, such as laminarin and fucoidan, remain largely unresolved [17,18]. Furthermore, several proposed signaling mechanisms are inferred from studies using purified ANE-derived polysaccharides or related elicitor systems rather than chemically characterized whole ANE formulations. The interpretation is further complicated by the multi-component nature of ANE, where interactions among metabolites can influence biological responses [26]. In addition, differences in extraction and processing methods contribute to variations in composition and bioactivity [30].

Another limitation is the weak connection between mechanistic studies and field observations. Although laboratory and greenhouse studies provide important information, field conditions introduce additional variables, including soil heterogeneity, microbial diversity, and environmental fluctuations that influence ANE performance [31,32]. Addressing these scales requires molecular, physiological, and ecological approaches that can link mechanistic responses to agronomic outcomes. A framework integrating ANE composition, signal perception, intracellular signaling, gene regulation, and plant responses is needed to improve understanding of ANE activity [33].

This review synthesizes the current knowledge on ANE by (i) describing the biochemical composition of *A. nodosum*, (ii) examining how extraction-dependent compositional variation influences ANE activity, (iii) examining molecular signaling pathways, (iv) linking these pathways with physiological responses, and (v) identifying key challenges and future research directions. This review presents a framework for understanding current understanding of the molecular processes associated with ANE-mediated regulation of plant growth, nutrient acquisition, and stress tolerance.

## 2. Biochemical Composition and Functional Constituents of ANE

The biological activity of ANE is derived from a heterogeneous mixture of bioactive metabolites, including structural polysaccharides, osmolytes, phenolic compounds, and trace phytohormone-like molecules that influence plant metabolism, cellular signaling, and stress responses [26]. Carbohydrates constitute the major biochemical fraction, representing 40–50% of the dry biomass, whereas ash, proteins, and lipids occur in lower proportions (14%, 3–4%, and 4–5%, respectively) [10,11]. Biological activity is influenced by structural characteristics, such as polymer size, branching pattern, molecular weight, and degree of sulfation, which affect biological activity and plant responses [34].

Structural polysaccharides are the most abundant and biologically important constituents of ANE. Alginates, which account for 20–30% of the dry biomass, are linear copolymers of β-D-mannuronic and α-L-guluronic acids with high water retention and cation-chelating properties. These properties improve soil moisture retention, rhizosphere ion exchange, and nutrient availability [35,36]. During extraction or partial hydrolysis, alginate-derived oligosaccharides are generated, which can act as elicitors associated with growth regulation and defense priming [26,35].

Laminarin, a β-(1→3)-glucan storage polysaccharide, occurs at concentrations ranging from 1 to 25% of dry biomass and functions as both a carbon reserve and a defense elicitor [15]. Studies using purified laminarin-derived oligosaccharides have shown association with activation of jasmonic acid (JA)- and salicylic acid (SA)-related signaling pathways involved in defense priming [23]. Purified β-glucan has also been reported to induce Ca^2+^ influx, ROS generation, and mitogen-activated protein kinase (MAPK) activation, linking elicitor perception to transcriptional responses associated with stress-responsive pathways [17,37]. However, these findings are primarily derived from studies using purified polysaccharides and should not be interpreted as direct evidence applicable to all ANE formulations.

Fucoidan is a sulfated fucose-rich heteropolysaccharide characterized by a high molecular weight and variable sulfation patterns, which influence its antioxidant capacity and stress-related responses [11,38]. Its bioactivity is affected by sulfate content, molecular size, monosaccharide composition, and chain conformation, which may influence biological activity and signaling-associated responses [34].

In addition to polysaccharides, ANE contains osmolytes, particularly mannitol, which constitutes 3–20% of dry biomass. Mannitol acts as a compatible solute involved in osmotic adjustment, membrane stabilization, and redox homeostasis under stress conditions [39]. Although not considered a major signaling molecule, it contributes to drought and salinity tolerance through interactions with stress-responsive metabolic pathways [16].

Phenolic compounds, particularly phlorotannins derived from phloroglucinol units, represent a major class of secondary metabolites in ANE. Total phenolic content typically ranges from 4 to 7 mg phloroglucinol equivalents g^−1^ dry biomass [40]. These compounds contribute to cellular redox balance by ROS scavenging and modulation of antioxidant enzyme systems [33,41]. Additional constituents, including carotenoids such as fucoxanthin and mineral nutrients such as Na, K, Mg, Ca, and P, further contribute to antioxidant activity and metabolic stability under stress conditions [15]. Although these mineral constituents contribute to the overall composition of ANE, their concentrations are generally insufficient to explain the biological responses reported for ANE, which are largely attributed to signaling- and regulation-associated effects [9].

ANE also contains low concentrations of phytohormone-like compounds, including auxins, cytokinins, and SA, which interact with endogenous hormonal networks involved in growth and stress responses [42]. Their role appears to be mainly regulatory, as ANE influences hormone biosynthesis, transport, and signaling sensitivity, rather than functioning as a direct hormone source [9].

In summary, ANE functions as a multi-component signaling system in which polysaccharides, osmolytes, phenolics, and hormone-like compounds collectively influence plant metabolism, stress responses, and physiological adaptation [26]. Variations in biomass sources, environmental conditions, and extraction methods contribute to the differences in composition and biological activities among studies [30]. The major classes of bioactive constituents, their occurrence, and functional roles are summarized in Table 1.

## 3. Extraction-Dependent Variation in ANE Composition and Mechanistic Consequences

The biological efficiency of ANE is highly influenced by the extraction methodology because extraction conditions determine the recovery, structural integrity, and relative abundance of bioactive constituents [48]. Variations in pH, temperature, solvent systems, and processing intensity can affect polymer size, sulfation degree, oxidation state, solubility, and oligosaccharide formation, thereby influencing the signaling potential and biological activity of ANE-derived compounds [18,34]. Consequently, mechanistic observations obtained using different ANE formulations may not be directly comparable, even when derived from the same source species.

Alkaline extraction is widely used for the recovery of structural polysaccharides, particularly alginates [50,51,52]. However, high pH and temperature may alter molecular weight distribution and reduce the abundance of thermolabile metabolites [19,34]. Consequently, alkaline extracts are generally enriched in high molecular weight polysaccharides, whereas lower molecular weight bioactive fractions may be reduced [30]. Such compositional differences may influence elicitor activity and downstream physiological responses.

Acid-based extraction is commonly used to recover sulfated polysaccharides, such as fucoidan and laminarin [44,45]. Although acid hydrolysis can increase polysaccharide recovery, it may also modify sulfation patterns and glycosidic linkages that contribute to biological activity [34,46]. Therefore, extraction efficiency alone is not necessarily indicative of biostimulant efficacy, as structural modifications can influence interactions with plant signaling systems.

Enzyme-assisted extraction (EAE) provides a milder alternative that facilitates the release of intracellular metabolites while preserving structural integrity. Compared with conventional chemical extraction, EAE generally maintains functional groups, antioxidant capacity, and oligosaccharide size distribution, which may contribute to greater consistency in biological responses [26,53,54].

Other approaches, including ultrasound-assisted, microwave-assisted, supercritical-fluid, and sequential biorefinery extraction systems, further modify the composition of recovered metabolites by altering cell disruption efficiency and selectivity toward particular compound classes [14,30,50,55]. These methods can influence the relative abundance of polysaccharides, phenolics, pigments, and other regulatory metabolites that may contribute to ANE-associated responses [53,54].

A major challenge in mechanistic studies is that extraction methodology can substantially alter the chemical composition of ANE formulations [19,30]. Variations in molecular weight distribution, sulfation patterns, polymer integrity, and oligosaccharide abundance may influence signaling-associated responses, including elicitor activity, gene-expression responses, and stress-related physiological processes [19,30,34,35]. Consequently, differences in plant responses among studies may partly reflect extraction-dependent compositional variation rather than intrinsic ANE activity [19,26,30].

The absence of standardized extraction protocols remains a significant limitation for establishing clear relationships among ANE composition, molecular responses, and agronomic performance [26]. Although extraction efficiency is frequently used to evaluate process performance, extracts with similar chemical yields may differ substantially in biological activity because structural characteristics often determine functional properties [19,30,35]. Future studies should integrate compositional characterization, bioassay-guided fractionation, and mechanistic analyses to establish clearer structure–function relationships and improve understanding of ANE-mediated signaling processes [30,36].

The major extraction methods and their functional implications are summarized in Table 2.

## 4. Molecular Mechanisms of ANE Action

Because ANE is a chemically complex mixture, mechanistic evidence has been generated using both whole ANE formulations and purified constituent fractions, particularly laminarin, fucoidan, and alginate-derived oligosaccharides. Throughout this section, findings obtained from purified compounds are discussed as mechanistic support for ANE-associated responses and should not be interpreted as direct evidence applicable to all ANE formulations. This distinction is important because the biological activity of whole extracts reflects the combined effects of multiple interacting constituents rather than individual fractions.

### 4.1. Signal Perception and Early Cellular Responses

Plant responses associated with ANE treatment are thought to involve the perception of bioactive constituents at the cell surface, followed by activation of intracellular signaling pathways [28,33]. However, the molecular mechanisms linking ANE perception to downstream signaling events remain incompletely resolved. ANE-derived metabolites, particularly laminarin, fucoidan, and alginate-derived oligosaccharides, have been reported to exhibit elicitor-like activity and are associated with defense- and stress-related signaling responses [9,35].

ANE-derived polysaccharides, particularly purified laminarin and related β-glucan fractions, have been proposed to act as microbe-associated molecular pattern (MAMP)-like or damage-associated molecular pattern (DAMP)-like signals that may be perceived by plasma membrane-localized pattern recognition receptors (PRRs) [17,58]. However, the receptors responsible for ANE perception remain largely unknown [27]. Studies on β-glucan perception have shown that laminarin is associated with activation of immune responses in grapevine, tobacco, *Arabidopsis*, barley, and *Nicotiana benthamiana* through receptor-like kinases (RLKs) and receptor-like proteins (RLPs), suggesting that related receptor classes could contribute to perception of ANE-associated polysaccharides, although direct evidence for ANE-specific ligand–receptor interactions remains unavailable [37,59].

Studies using ANE formulations and purified ANE-derived polysaccharides have reported rapid cellular responses, including cytosolic Ca^2+^ influx, membrane depolarization, extracellular alkalinization, ion flux changes, ROS generation, and MAPK-associated signaling events, which are characteristic features of early defense responses [60,61]. Although Ca^2+^ influx, ROS generation, and MAPK activation are consistently associated with ANE-mediated responses, the temporal sequence and relationships among these events remain unresolved [28,33,60]. Current evidence suggests that these components function as interconnected regulatory modules with feedback regulation and pathway crosstalk rather than as a strictly linear signaling pathway [28,37,61]. In grapevine cells, laminarin rapidly induces Ca^2+^ influx, oxidative bursts, alkalinization, and MAPK activation within minutes of elicitor exposure [37,59].

Calcium signaling is frequently associated with elicitor-mediated responses and is considered an important component of the signaling networks linked to ANE-associated activity [33], with cytosolic Ca^2+^ acting as a second messenger decoded by Ca^2+^-dependent protein kinases (CDPKs), calmodulin, and calcineurin B-like proteins that coordinate downstream phosphorylation cascades and transcriptional responses [33]. Based on current understanding of plant elicitor signaling systems, Ca^2+^ influx may involve cyclic nucleotide-gated, glutamate receptor-like, and mechanosensitive channels, although their specific roles in ANE-mediated responses remain unresolved. Signal intensity is also influenced by ligand structure; in *N. benthamiana*, laminarin induces stronger Ca^2+^ and ROS responses than laminarihexaose, indicating that oligosaccharide size and conformation influence signaling efficiency [59].

ROS production is frequently associated with Ca^2+^-dependent signaling processes and may contribute to signal amplification and defense-related responses [61]. ANE-associated physiological responses appear to be influenced by the balance between ROS generation and antioxidant scavenging systems [61]. Major ROS sources include respiratory burst oxidase homologs (RBOHs), apoplastic peroxidases, and electron transport chains, whereas antioxidant systems, including superoxide dismutase, catalase, and the ascorbate–glutathione cycle, maintain redox homeostasis [16,61]. Membrane depolarization and changes in ion flux further contribute to signal propagation [60].

Available evidence suggests that these early signaling events may converge on MAPK-associated pathways, which integrate Ca^2+^, ROS, and hormone-associated signals to regulate downstream gene expression [28,37]. Laminarin-induced MAPK activation is associated with increased expression of defense-related genes, enhanced chitinase and β-1,3-glucanase activity, and accumulation of phytoalexins, such as resveratrol and ε-viniferin [17]. However, the extent to which these responses occur in chemically diverse ANE formulations remains unclear. In plant signaling systems, MAPK modules are known to phosphorylate transcription factors belonging to the WRKY, MYB, NAC, and AP2/ERF families; however, direct evidence linking specific MAPK modules to ANE-mediated responses remains limited [33].

The physiological effects of ANE are due to interactions among multiple signaling networks rather than the action of individual metabolites [26]. Variations in extraction conditions and chemical composition can further modify these responses by altering polysaccharide structure and metabolite abundance [19,34]. Importantly, much of the mechanistic evidence discussed above originates from studies using purified laminarin, fucoidan, β-glucans, or related elicitor systems rather than chemically defined whole ANE formulations. Consequently, extrapolation of these mechanisms to all ANE products should be interpreted cautiously, particularly because ANE formulations may differ substantially in composition owing to extraction methodology, biomass source, and processing conditions.

**Figure 1:**
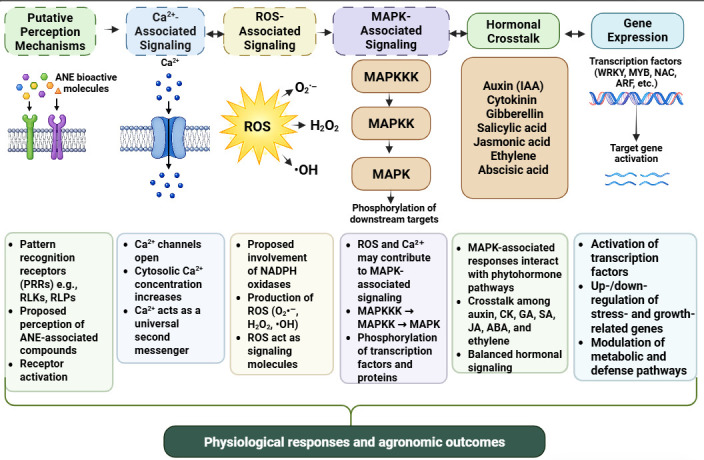
Proposed working framework summarizing signaling and regulatory processes associated with ANE-mediated plant responses. Several components are supported by studies using whole ANE formulations, whereas others are inferred primarily from studies employing purified ANE-derived polysaccharides (e.g., laminarin and fucoidan) or related elicitor systems. Interactions among perception mechanisms, Ca^2+^-associated signaling, ROS-associated signaling, MAPK-associated signaling, hormonal networks, and transcriptional regulation are influenced by feedback regulation, pathway crosstalk, developmental stage, extract composition, and environmental conditions. Consequently, the proposed relationships among signaling components should not be interpreted as universally established for all ANE formulations or as a fully resolved signaling cascade. Solid borders indicate processes supported by whole ANE studies, whereas dashed borders indicate processes inferred primarily from purified ANE-derived polysaccharides or related elicitor systems. The figure is intended as a conceptual framework summarizing current evidence and knowledge gaps rather than a definitive mechanistic model.

Despite increasing evidence, major gaps remain in receptor identification, ligand specificity, and interactions among co-occurring metabolites [27,58]. Addressing these limitations will require fractionation-based assays, receptor-binding studies, phosphoproteomics, Ca^2+^ imaging, and integrated multi-omics approaches to establish clearer structure–activity relationships and improve the consistency of formulations [28,33]. Collectively, these signaling and regulatory interactions provide a conceptual framework linking ANE composition with downstream molecular and physiological responses discussed in this review. The major signaling pathways involved in ANE-mediated molecular responses are shown in Figure 1.

### 4.2. Hormonal Crosstalk and Growth Regulation

The growth-promoting effects of ANE are primarily associated with the modulation of endogenous phytohormonal pathways rather than direct nutrient supply [9,42]. Chemical and metabolomic analyses have identified phytohormone-like compounds, including indole-3-acetic acid (IAA), cytokinins, gibberellins (GA), and betaines, along with polysaccharides and phenolic compounds that may interact with plant signaling networks involved in growth and stress adaptation [62]. Reported concentrations (IAA: 0.5–4.3 µg g^−1^; cytokinins: ~15–75 ng g^−1^) indicate the presence of low but biologically active levels of these compounds [63]. However, the concentrations detected in ANE are generally insufficient to explain the observed physiological responses solely through direct hormone supply. Consequently, current evidence suggests that ANE primarily influences plant development through modulation of endogenous hormone biosynthesis, transport, sensitivity, and signal transduction pathways [42,64].

Modulation of auxin-associated pathways is among the most consistently reported responses to ANE treatment. Upregulation of auxin-responsive genes and signaling components has been associated with increased root length, plant height, and leaf number in tomato, as well as improved root growth and yield under water-deficit conditions [20,29]. These effects are consistent with established roles of auxin in cell elongation, root initiation, developmental plasticity, and nutrient acquisition [65].

Transcriptomic and physiological studies further suggest involvement of cytokinin-associated pathways in ANE-mediated growth responses. Cytokinins regulate cell division, meristem activity, and nutrient allocation, whereas interactions between auxin and cytokinin signaling influence root–shoot balance and source–sink relationships [29,64]. Consequently, ANE-associated modulation of these pathways may contribute to coordinated regulation of plant architecture and biomass distribution [29,64].

Findings from gene-expression and physiological studies indicate that ANE treatment may also influence GA-associated pathways involved in stem elongation and reproductive development. In ANE-treated plants, GA responses interact with stress-related hormonal pathways, particularly abscisic acid (ABA), which is involved in drought, salinity, and nutrient stress responses [16,66]. ANE treatment has been associated with maintenance of growth under stress conditions through effects on ABA-related processes, such as stomatal regulation and osmotic adjustment. Partial alleviation of ABA-mediated growth inhibition has been proposed as one mechanism contributing to sustained growth in adverse environments [67].

Defense-related hormones, including jasmonic acid (JA), salicylic acid (SA), and ethylene (ET), have also been implicated in ANE-associated responses. Although growth and defense pathways are often antagonistic, ANE appears to support both processes by promoting a primed physiological state rather than continuous defense activation, enabling faster stress responses with limited effects on growth [68,69]. However, evidence supporting these interactions is derived from a combination of whole ANE studies, transcriptomic analyses, and studies using ANE-derived polysaccharides, and the relative contribution of individual hormonal pathways remains incompletely resolved.

Overall, ANE appears to influence multiple interacting hormonal pathways rather than a single hormonal route. The balance among growth-, stress-, and defense-related hormones is likely to contribute to the diverse physiological responses reported following ANE applications [62]. Nevertheless, causal relationships between specific ANE constituents and individual hormonal signaling pathways remain largely unresolved and require further mechanistic investigation.

### 4.3. Transcriptional Reprogramming and Gene Regulatory Pathways

ANE treatment has been associated with widespread transcriptional reprogramming through hormone- and elicitor-responsive regulatory networks linked to growth, metabolism, and stress adaptation [28,62,64]. Transcriptomic studies have consistently demonstrated differential expression of genes associated with plant growth, primary metabolism, nutrient acquisition, and stress responses following ANE treatment [28,70]. Among the most frequently reported changes are the upregulation of auxin-responsive genes, including AUX/IAA and ARF transcription factors, as well as genes involved in cell elongation, root development, cell wall remodeling, and nutrient transport [29,71].

In addition to auxin-responsive genes, cytokinin-responsive genes associated with cell cycle progression, meristem activity, and nutrient allocation have been reported to be differentially expressed following ANE treatment [29,64]. Transcriptomic evidence also indicates modulation of GA- and ABA-responsive regulatory pathways involved in stem development, stomatal regulation, osmotic adjustment, and stress adaptation [16,66,72].

Transcriptomic and gene-expression studies further suggest involvement of defense-associated transcriptional pathways linked to JA, SA, and ET signaling following ANE application [68,73]. These pathways regulate genes encoding pathogenesis-related proteins, antioxidant enzymes, and enzymes involved in secondary metabolite biosynthesis. As a result, these observations are consistent with the establishment of a primed transcriptional state that may enable rapid defense activation following stress exposure while minimizing constitutive metabolic costs.

Many of these transcriptional responses involve major transcription factor families, including WRKY, MYB, NAC, AP2/ERF, ARF, and EIN3/EIL proteins, which function as central integration nodes for hormonal and redox signaling. MAPK-associated signaling has been proposed to contribute to transcriptional regulation through phosphorylation of transcription factors and modulation of downstream gene expression, although direct evidence for specific MAPK–transcription factor relationships in ANE-mediated responses remains limited [37]. Furthermore, much of the available evidence is based on transcriptomic associations, and the upstream signaling events linking ANE perception to specific transcriptional responses remain incompletely resolved.

Overall, ANE is associated with the modulation of a highly interconnected gene regulatory network rather than isolated transcriptional responses. The transcriptional profile varies according to developmental stage, extract composition, and environmental conditions [28,70]. Notably, most available evidence is derived from transcriptomic analyses that identify associations between ANE treatment and differential gene expression, whereas causal relationships between specific ANE constituents and individual regulatory pathways remain incompletely resolved. Future studies integrating high-resolution transcriptomics, temporal analyses, and structure–function approaches are required to distinguish primary transcriptional events from downstream responses and establish clearer mechanistic relationships.

### 4.4. Stress Signaling and Defense Activation

ANE has been reported to function as a defense-associated biostimulant and is frequently associated with enhanced endogenous stress and immune responses [17,35]. Instead of directly targeting pathogens, ANE is proposed to promote a primed physiological state that enables faster and stronger defense responses after stress exposure [23,68]. This effect has been largely attributed to elicitor-active polysaccharides, particularly laminarin and fucoidan, which have been associated with activation of plant immune responses [27,44]. Priming may enhance defense capacity while minimizing constitutive metabolic costs [68]. ANE treatment has been associated with rapid defense-related responses, including ROS production and maintenance of redox homeostasis [44,58,61]. These processes may contribute to signal amplification, antimicrobial activity, and protection against oxidative damage during stress.

Studies from controlled-environment and field systems suggest that ANE application may contribute to induced systemic resistance (ISR)-like responses and enhanced protection against pathogens [74,75]. In grapevines, ANE reduced downy mildew incidence from 70% to 40–50%, and further to 22% when combined with beneficial microorganisms, demonstrating enhanced disease suppression under integrated management systems [76]. These observations are consistent with a role for ANE in enhancing plant defense capacity across different pathosystems.

In addition to disease suppression, ANE treatment has been associated with increased activity of several defense-related enzymes. In arugula microgreens, PAL, catalase, superoxide dismutase, and polyphenol oxidase activities increased following ANE application, accompanied by higher phenolic and flavonoid accumulation [77]. Similar increases in chitinase, β-1,3-glucanase, and peroxidase activities have been reported in tomato and pepper [69,75]. Enhanced phenylpropanoid metabolism is generally associated with cell wall reinforcement and production of antimicrobial metabolites [60].

ANE treatment has also been associated with increased expression of defense-related genes, including PR1, PR5, and NPR1, and the modulation of pathways involved in plant immunity [73]. Importantly, enhanced defense responses are frequently accompanied by continued growth and biomass accumulation, supporting the hypothesis that ANE may promote defense priming without substantial growth penalties [20,68]. However, the extent to which these responses are consistently reproduced across different ANE formulations and crop systems remains uncertain.

However, the molecular basis of ANE-mediated defense priming remains incompletely understood. Much of the mechanistic evidence originates from studies using purified ANE-derived polysaccharides or related elicitor systems, whereas direct evidence linking specific ANE constituents to defense signaling networks remains limited. Despite increasing evidence for ANE-associated defense responses, major gaps remain in receptor identification, signaling hierarchy, and formulation-dependent variability [27]. Future studies should combine biochemical, molecular, and field-based approaches to establish defense signatures, validate mechanisms, and improve prediction of ANE-associated defense responses under diverse agricultural conditions.

### 4.5. Modulation of Root Architecture and Nutrient Uptake

The root system is the primary interface for water and nutrient acquisition, and its structure strongly influences nutrient-use efficiency and crop productivity [9]. ANE has been associated with morphological and physiological changes in root systems that may enhance soil exploration and nutrient acquisition [65,71]. ANE application has been reported to increase primary root elongation, lateral root formation, and root hair development, thereby increasing root surface area and absorptive capacity. In maize, ANE was associated with increased root length and biomass together with improved Mg, B, Zn, and Mn uptake under P-limited conditions [22]. Similar responses have been reported in tomato and eggplant seedlings [78,79]. Anatomical changes, including cortical expansion and enhanced vascular differentiation, have been observed and may contribute to improved transport efficiency under both optimal and stress conditions [80].

Lateral root formation is among the most consistently reported root responses to ANE treatment. In *Arabidopsis*, ANE increased root branching and elongation [81], and similar effects have been observed in cereal crops [82]. ANE treatment has also been associated with increased root hair initiation and elongation, potentially enhancing root–soil contact and acquisition of relatively immobile nutrients, such as phosphorus (P) [83]. Consequently, increased P-accumulation has been observed under P-deficient conditions [22].

In addition to structural changes, ANE treatment has been associated with modulation of nutrient transporter genes and nutrient assimilation pathways. Upregulation of nitrate transporters, including BnNRT1.1 and BnNRT2.1, and sulfate transporters, such as BnSultr4.1, has been reported following ANE treatment [20], indicating an enhanced nutrient uptake capacity. ANE treatment has also been associated with increased expression of gene encoding key nitrogen assimilation enzymes, including nitrate reductase (NR), nitrite reductase (NiR), glutamine synthetase (GS), and glutamate synthase (GOGAT) [84]. In cereals, increased nitrate uptake under reduced fertilizer input further supports coordinated effects of nutrient uptake and assimilation [85].

P-acquisition has additionally been associated with increased expression of regulatory genes, such as ZmPHR1 and PTF1, which are involved in P-sensing and homeostasis under deficient conditions [22]. ANE treatment has also been linked to improved uptake of K, Ca, Mg, Zn, Mn, and Fe, contributing to ionic balance, metabolic stability, and maintenance of photosynthetic performance under stress [86,87]. These observations are generally interpreted as consequences of ANE-associated modulation of root development, nutrient transport systems, and regulatory pathways rather than direct nutrient contributions from the extract itself.

In summary, ANE appears to enhance nutrient-use efficiency through coordinated effects on root architecture, nutrient transport, and assimilation pathways [88]. However, the relative contributions of extract-derived bioactive compounds, and endogenous hormonal regulation, and transcriptional reprogramming remain incompletely resolved. Furthermore, variability in extract composition and application conditions contributes to inconsistent outcomes among studies. Future research integrating root phenotyping, transcriptomics, transporter activity analyses, functional genomics, and isotope-based nutrient tracing will be required to establish clearer mechanistic relationships between ANE-associated root development and nutrient acquisition.

### 4.6. ANE-Mediated Rhizosphere Microbiome Interactions

The rhizosphere is a dynamic interface where plant roots interact with diverse microbial communities that influence nutrient cycling, plant health, and stress resilience [89]. In addition to direct effects on plant signaling and root development, ANE treatment has been associated with modifications in rhizosphere microbial communities through changes in root architecture, root exudation patterns, and the availability of bioactive metabolites [65,83]. Current evidence suggests that microbiome-associated effects are likely mediated through ANE-induced changes in plant physiology and root-derived signals rather than through direct microbial targeting mechanisms.

Sequencing-based studies have shown that ANE application is associated with enrichment of beneficial taxa, including *Rhizobium*, *Bradyrhizobium*, and *Sphingomonas*, which are linked to plant growth promotion and nutrient cycling [24]. Increased abundance of plant growth-promoting rhizobacteria (PGPR), including *Pseudoxanthomonas*, *Novosphingobium*, and *Kitasatospora*, has also been reported following ANE treatment [25]. In legumes, ANE application has been associated with enhanced nodulation and rhizobial diversity, indicating the improved selection of nitrogen-fixing symbionts [90].

Changes in microbial community composition are accompanied by functional changes in microbial activity. Root exudates from ANE-treated plants have been reported to enhance chemotaxis and metabolic activity in beneficial bacteria, such as *Pseudomonas protegens*, including expression of genes linked with antimicrobial compound production and nutrient acquisition [25]. In addition, ANE-derived polysaccharides may function as prebiotic substrates that selectively stimulate carbohydrate-utilizing microbial populations [34]. These observations suggest that ANE-associated effects on plant metabolism may indirectly influence microbial community structure and activity within the rhizosphere [91].

Microbiome shifts related to ANE treatment have been associated with nutrient acquisition and disease suppression. For example, enrichment of taxa such as *Mortierella* spp. has been associated with improved nutrient availability and reduced pathogen incidence [24]. In addition, ANE treatment has also been linked to changes in root metabolite profiles, including benzoxazinoids such as DIMBOA and MBOA, which may further influence microbial selection and community composition [25]. These findings support the concept that ANE-mediated transcriptional and metabolic reprogramming can extend beyond the plant itself to influence plant–microbe interactions in the rhizosphere.

Despite increasing evidence of microbiome modulation, the relationships between specific ANE constituents, root signaling pathways, exudate composition, and microbial selection remain poorly understood because most available studies are correlative and lack functional validation. Environmental variability and soil heterogeneity further constrain reproducibility under field conditions [30]. Future studies integrating synthetic microbial communities, root exudate metabolomics, and field-scale validation will be required to establish mechanistic relationships and determine the contribution of microbiome-mediated processes to ANE-associated plant responses. Table 3 summarizes the principal molecular pathways, representative markers, and evidence supporting ANE-associated responses, highlighting the integration of signaling, transcriptional, and physiological processes underlying plant growth, nutrient acquisition, and stress adaptation.

## 5. Agronomic Outcomes: Linking Molecular Mechanisms to Plant Performance

The application of ANE enhances plant growth, yield, and quality across diverse crop systems. Importantly, these agronomic responses are considered downstream consequences of ANE-mediated signaling, transcriptional reprogramming, hormonal modulation, and metabolic adjustment rather than direct nutrient supplementation [9,94]. The modulation of hormone-associated pathways, photosynthetic processes, nutrient transport, and primary metabolism is frequently associated with enhanced vegetative growth, including increased plant height, leaf area, root development, and biomass accumulation [20,65,71,95]. These responses are associated with improved photosynthetic performance, nutrient-use efficiency, and primary metabolic processes [65,71].

Enhanced nutrient acquisition, maintenance of physiological homeostasis, and improved stress resilience may contribute to increased reproductive performance and yield across multiple crop systems [16,66]. However, yield responses vary substantially among studies and are influenced by genotype, environmental conditions, crop management practices, extract composition, and application strategy [43,96,97]. ANE treatment has also been associated with improvements in crop quality traits, including increased accumulation of soluble solids, antioxidants, phenolics, flavonoids, and other secondary metabolites [74,98]. These responses are associated with changes in secondary metabolism and enhanced antioxidant capacity [40].

Many agronomic benefits of ANE become particularly evident under abiotic stress conditions, including drought, salinity, and nutrient limitation. Under water-deficit conditions, controlled environment and greenhouse studies have shown that treated plants maintain higher photosynthetic activity, chlorophyll content, and biomass accumulation [93]. Seed priming and foliar application have also been associated with improved germination and seedling vigor relative to untreated plants [78]. These responses are associated with maintenance of redox homeostasis, osmotic adjustment, and hormonal balance [16].

Although ANE applications frequently improve plant performance, the responses are not universally positive and may vary depending on the formulation characteristics, application strategy, crop genotype, and environmental conditions. Variability in ANE composition arising from extraction methodology and formulation characteristics can influence the magnitude of molecular, physiological, and agronomic responses [19,30,43]. In addition, responses observed under controlled environments are not always reproduced under field conditions, where environmental variability and plant–soil–microbe interactions introduce additional complexity [31,32]. Dose- and formulation-dependent responses further indicate that treatment efficacy is strongly context dependent [43,94]. Consequently, agronomic outcomes should be interpreted as emergent properties arising from interactions among formulation characteristics, plant regulatory responses, and environmental conditions rather than as direct and universally predictable effects of ANE application.

Collectively, these observations support the view that agronomic performance represents the integrated outcome of ANE-mediated molecular, transcriptional, physiological, and metabolic responses discussed in the preceding sections. Nevertheless, improved standardization of formulations and multi-environment validation remain necessary to strengthen mechanistic interpretation and improve prediction of field performance [30,94].

Table 4 illustrates how molecular and physiological responses are translated into agronomic outcomes across different crop systems, applications, and production environments.

## 6. Integrated Functional Pathway of ANE Action

The physiological and agronomic effects of ANE can be interpreted using an integrated framework linking bioactive compound perception, intracellular signaling, transcriptional regulation, and plant performance. ANE is generally considered to function as a multi-component signaling system that modulates interconnected pathways at the cellular, tissue, and whole-plant levels. Transcriptomic and metabolomic studies have demonstrated simultaneous changes in growth, defense, nutrient metabolism, and stress-related processes following ANE application, supporting a network-based model rather than effects attributable to individual metabolites [28,102].

As discussed in previous sections, ANE-associated responses are proposed to involve perception of bioactive constituents, activation of interconnected signaling networks, transcriptional reprogramming, and downstream physiological adjustments. However, many components of this framework remain incompletely resolved, particularly receptor identity, signaling hierarchy, and the relative contribution of individual ANE constituents [18,27].

Current evidence is consistent with a systems-level model in which signaling-associated responses interact with hormonal, transcriptional, metabolic, and redox regulatory networks to coordinate nutrient metabolism, growth, defense responses, and stress adaptation [16,28].

At the physiological level, these molecular changes manifest as altered root architecture, enhanced nutrient uptake, and improved photosynthetic performance, thereby translating molecular responses into measurable plant traits [65,71]. These responses are frequently associated with increased biomass accumulation, improved nutrient-use efficiency, and enhanced physiological resilience under both optimal and stress conditions [22,87].

At the rhizosphere level, ANE-induced changes in root development, root exudation, and plant metabolism may influence microbial community composition and activity, extending ANE-associated responses beyond plant physiology to plant–microbe interactions [24,83]. Although increasing evidence supports microbiome involvement, the magnitude and consistency of these effects remain dependent on environmental conditions and require further validation [102].

Collectively, available evidence suggests that ANE functions primarily as a signaling modulator that integrates molecular, physiological, and ecological processes across multiple biological scales. Rather than acting principally as a nutrient source, ANE appears to influence endogenous regulatory networks that contribute to growth, nutrient acquisition, stress adaptation, and plant–microbe interactions. Nevertheless, the proposed framework should be regarded as a working model because several mechanistic links remain inferred from purified ANE-derived polysaccharides, transcriptomic associations, or related elicitor systems and require further experimental validation [32,68].

## 7. Challenges and Limitations

### 7.1. Variability in Extract Composition and Lack of Standardization

Variations in ANE composition remain a major challenge for reproducibility and agronomic consistency. Compositional heterogeneity arises from the extraction methodology, seasonal harvesting, geographic origin, processing conditions, environmental growth factors, and intrinsic biological variation within the source biomass [12,47,48,103]. Consequently, extracts derived from the same species frequently differ in chemical composition and biological activity, reducing comparability across studies and complicating mechanistic interpretation [43].

Extraction parameters, including temperature, pH, solvent system, and extraction duration, significantly influence major polysaccharides such as alginates, laminarin, and fucoidan [47]. These conditions affect the molecular weight distribution, sulfation degree, chain conformation, and polymer integrity, all of which may influence biological activity and signaling-associated responses.

Variability in monosaccharide composition, sulfation patterns, and other structural features further contributes to differences in charge density, molecular interactions, and biological activity [46,104]. Because many proposed ANE-associated responses depend on perception and signaling processes, even subtle structural differences may influence receptor recognition, elicitor activity, transcriptional responses, and downstream physiological outcomes [27]. However, the absence of identified ANE-specific receptors currently limits direct assessment of structure–activity relationships and complicates interpretation of extraction-dependent effects.

Consequently, extracts derived from the same species can produce substantially different biological effects, even at comparable application rates, indicating that ANE efficacy depends not only on concentration but also on molecular composition and structural characteristics [43]. This variability complicates dose–response interpretation, limits cross-study comparisons, and remains a major obstacle to establishing clear structure–function relationships and reproducible mechanistic models. Future studies should combine detailed compositional characterization, bioassay-guided fractionation, and mechanistic validation to establish more robust links between extract composition and biological activity.

### 7.2. Limited Understanding of Molecular Mechanisms

Despite significant advances in characterizing the physiological, biochemical, and transcriptional responses associated with ANE, key aspects of ANE perception and signal transduction remain unclear [27,105]. Seaweed-derived polysaccharides, including laminarin, fucoidan, and alginate-derived oligosaccharides, have been widely proposed as elicitors. However, the receptors responsible for their perception remain unidentified, preventing the establishment of direct causal links between specific ANE components and downstream biological responses.

In plants, elicitor perception is typically mediated by plasma membrane-localized pattern recognition receptors (PRRs). However, no ANE-specific receptor–ligand interactions have been conclusively demonstrated [105]. Receptor-like kinases (RLKs) and lectin receptor kinases have been proposed as potential candidate receptors; however, direct evidence of ligand binding, receptor activation, and signaling specificity remains unavailable. Consequently, the structural determinants governing receptor recognition, including sulfation patterns, polymer size, glycosidic linkages, and molecular conformation, remain poorly understood [27].

Early cellular responses, including cytosolic Ca^2+^ influx and ROS generation, membrane depolarization, and kinase activation, have been reported following treatment with ANE formulations or purified ANE-derived polysaccharides [18,37]. Studies using purified laminarin support the involvement of Ca^2+^-dependent signaling pathways; however, the extent to which these mechanisms apply across chemically diverse ANE formulations remains unclear. However, the ion channels, Ca^2+^ sensors, and immediate downstream targets involved ANE-associated responses remain poorly characterized [18].

MAPK-associated signaling and enrichment of hormone- and stress-responsive pathways have been reported in ANE-treated systems and transcriptomic datasets [92]. However, the identities, regulatory relationships, and functional roles of specific MAPK modules remain unresolved [37].

ANE-associated responses share several characteristics with pattern-triggered immunity (PTI), including the activation of defense enzymes, pathogenesis-related proteins, and cell wall-associated pathways [92]. Nevertheless, it remains unclear whether these responses represent canonical PTI, modified PTI-like signaling, or distinct regulatory networks, highlighting the limited resolution of current immune-signaling models.

Although MAPK-associated and hormone-responsive pathways are consistently implicated in ANE responses, the absence of receptor-level evidence prevents the reconstruction of complete signaling networks [106]. Pathway redundancy, feedback regulation, and the multi-component nature of ANE further complicate biological interpretation and limit structure–activity relationship (SAR) analyses [107]. In addition, much of the available mechanistic evidence is derived from purified ANE-derived polysaccharides or related elicitor systems rather than chemically characterized whole ANE formulations, making extrapolation across products difficult. Consequently, many currently proposed mechanistic frameworks should be regarded as working hypotheses rather than fully validated signaling models.

Future research should prioritize receptor identification, fractionation-based bioassays, ligand-binding studies, genetic validation, phosphoproteomics, and integrated multi-omics approaches to elucidate the mechanisms underlying ANE perception and signaling. These approaches are essential for establishing causal relationships between molecular structures, signaling pathways, and phenotypic outcomes.

### 7.3. Complexity of Multi-Component Interactions

ANE contains diverse bioactive constituents, including polysaccharides, phenolic compounds, osmoprotectants, minerals, and phytohormone-like molecules. This compositional diversity underpins the wide range of responses observed across crops and environmental conditions, indicating that ANE functions as a multi-component regulatory system rather than through the activity of individual constituents [26,33,49,56].

Evidence from physiological, transcriptomic, and metabolomic studies suggests that ANE-mediated responses arise from the coordinated activity of multiple interacting constituents rather than a single dominant bioactive compound [28,99]. Consequently, growth promotion, stress adaptation, and nutrient-acquisition responses are likely to reflect emergent properties of complex regulatory interactions [108,109].

Functionally, ANE has been associated with the modulation of multiple hormonal pathways, including auxin, cytokinin, JA, and SA signaling [49]. These responses are accompanied by the accumulation of stress-associated metabolites, such as proline and soluble sugars, which contribute to osmotic adjustment and membrane stabilization [99]. Interactions among ANE constituents may be synergistic, additive, or antagonistic, depending on their concentration, structural properties, and environmental context, thereby influencing the overall biological activity [26]. However, direct experimental evidence quantifying these interaction effects remains limited.

Most studies continue to rely on whole-extract applications rather than chemically defined fractions, limiting attribution of biological responses to specific compounds or metabolite combinations [30]. Although fucoidans, alginates, and mannitol are recognized as major functional fractions, their individual contributions and interaction dynamics remain poorly understood [34,110]. Consequently, causal relationships between individual ANE constituents and specific molecular responses remain difficult to establish. This limitation further complicates the interpretation of receptor-mediated signaling, transcriptional regulation, and downstream physiological responses discussed throughout this review.

This biochemical complexity remains a major obstacle to mechanistic resolution, structure–function analysis, and rational formulation design [26]. Future studies should integrate bioassay-guided fractionation, factorial mixture designs, and network-based modeling approaches to identify functionally active combinations and quantify their interaction effects [33].

### 7.4. Inconsistencies in Experimental Design and Reporting

The current literature on ANE exhibits substantial variability in experimental design, limiting direct comparisons across studies and reducing the reliability of mechanistic interpretation [111]. Studies differ in extract concentration, application frequency, delivery method, crop species, genotype, developmental stage, and environmental conditions [94]. This heterogeneity complicates the identification of reproducible biological responses and constrains the development of unified mechanistic models.

Reported application rates vary substantially among studies [43,99]. However, nominal concentration alone does not adequately reflect biological exposure because plant responses are influenced by application timing, frequency, formulation characteristics, and developmental stage [56]. As a result, similar application rates may produce different molecular, physiological, and agronomic responses across experimental systems.

Environmental variables, including soil fertility, irrigation regime, nutrient availability, and stress intensity, further influence plant performance and contribute to variations among experiments [19,48]. These factors complicate the establishment of reproducible dose–response relationships and limit cross-study integration. Environmental variation may also influence signaling-associated responses, transcriptional regulation, and stress-adaptation pathways, further complicating mechanistic comparisons among studies.

A major limitation is the inadequate reporting of the extract composition and processing history. Many studies do not quantify major bioactive constituents, including alginates, laminarins, phenolic compounds, and mannitol [9], thereby limiting their reproducibility and preventing comparisons among formulations [48]. Consequently, products marketed under similar labels may differ substantially in chemical composition and biological activity.

Incomplete reporting of extraction procedures further complicates interpretation because compositional differences arising from processing conditions may influence signaling-associated responses and downstream physiological outcomes [19,56]. Consequently, observed variation among studies may reflect methodological differences rather than biological effects alone.

Collectively, variability in experimental design, extract characterization, and reporting standards remains a major obstacle to reproducibility, mechanistic validation, and predictive interpretation of ANE-mediated responses. Without standardized reporting of formulation composition and experimental conditions, it remains difficult to distinguish genuine biological mechanisms from context-dependent experimental effects. Future studies should adopt harmonized reporting frameworks that incorporate detailed extract characterization, dose normalization, crop genotype, environmental conditions, application timing, and standardized response metrics to improve reproducibility and facilitate cross-study integration [56,94].

### 7.5. Field–Laboratory Discrepancies

A persistent limitation in ANE research is the discrepancy between responses observed under controlled experimental conditions and field performance, which constrains the translation of molecular insights into reliable agronomic predictions [112,113]. Controlled studies permit the precise regulation of environmental variables, including temperature, photoperiod, irrigation, and nutrient availability, facilitating a detailed investigation of signaling pathways, transcriptional dynamics, and biochemical responses [114,115]. However, such systems do not fully represent field environments, where plants are exposed to multiple interacting stresses, spatial soil heterogeneity, and fluctuating climatic conditions [116,117].

Field-grown plants also interact with complex soil and phyllosphere microbial communities that influence nutrient availability, hormonal regulation, and stress adaptation [118,119]. Because these interactions are often simplified or absent under controlled conditions, molecular responses identified in laboratory studies may not fully predict field performance. Consequently, physiological improvements observed in laboratory and greenhouse studies, including enhanced photosynthetic efficiency and biomass accumulation, often exhibit reduced magnitude or greater variability under field conditions [120]. These observations indicate that ANE efficacy is strongly context dependent. The differences between controlled and field studies likely reflect system-level biological complexity rather than inconsistencies in the intrinsic activity of ANE. Interactions among soil properties, microbial communities, and environmental conditions can substantially modify plant responses under field conditions [121].

Genotype × environment × management (G × E × M) interactions further contribute to variability across locations and growing seasons, generating site-specific responses that are difficult to generalize [31,122]. Such interactions complicate extrapolation of mechanistic findings across production systems and reduce predictive reliability. Consequently, signaling-associated, transcriptional, and physiological responses observed under controlled conditions may not always translate directly into equivalent agronomic outcomes under field environments.

These limitations highlight the need for research frameworks that directly connect molecular, physiological, microbiome, and agronomic datasets across environmental scales [32]. Bridging this gap is essential for validating proposed mechanistic models and determining which molecular responses are consistently associated with field-level performance. Future studies should integrate omics-based analyses, environmental monitoring, microbiome profiling, and agronomic assessments within multilocation field trials to validate mechanistic hypotheses, identify major sources of variability, and improve the prediction of ANE performance under realistic agricultural conditions.

### 7.6. Limited Integration of Multi-Omics Approaches

Transcriptomic studies have provided important insights into ANE-associated changes in gene expression related to nutrient transport, metabolism, hormone signaling, and stress responses. In tomatoes, ANE treatment induces extensive differential gene expression, with the enrichment of pathways associated with carbon metabolism, MAPK signaling, phenylpropanoid biosynthesis, and hormonal regulation [28]. In Arabidopsis, ANE modulates numerous stress-responsive genes under salinity stress, and different ANE formulations produce distinct transcriptional responses, highlighting formulation-dependent variability [64,70]. Despite these advances, transcriptomics alone provides an incomplete representation of ANE-mediated responses because transcriptional changes do not necessarily correspond to protein abundance, signaling activity, or physiological function.

Agronomic and physiological responses are ultimately determined by protein activity, metabolic flux, and signaling dynamics, all of which extend beyond transcript abundance [123]. For example, ANE-associated stress tolerance has been linked to the accumulation of compatible solutes, antioxidant metabolites, and other biochemical adjustments that are not fully captured by transcriptomic datasets alone [124,125,126].

Although ANE enhances osmoprotectant accumulation, antioxidant activity, and phenolic metabolism, these responses have rarely been integrated with proteomic or phosphoproteomic datasets within the same experimental pathway. This remains a major limitation because key regulatory pathways, including MAPK-associated and hormone-signaling networks, are predominantly regulated through protein phosphorylation and other post-translational modifications [37]. Without protein-level analyses, critical regulatory nodes and signaling relationships remain difficult to resolve.

Furthermore, ionomic and microbiome datasets are seldom integrated with transcriptomic analyses, despite their central roles in nutrient acquisition, stress adaptation, and rhizosphere regulation [24,83]. The absence of cross-scale integration limits our understanding of how molecular regulation interacts with nutrient dynamics, microbial interactions, and environmental conditions, resulting in fragmented mechanistic interpretations [127]. This limitation also hinders validation of the integrated framework proposed in this review, in which signaling, transcriptional, physiological, and microbiome-associated responses are interconnected across multiple biological scales.

Future studies should integrate transcriptomics, proteomics, metabolomics, phosphoproteomics, ionomics, and microbiome profiling to identify regulatory hubs, establish causal relationships, and connect molecular responses with physiological performance under both controlled and field conditions [33,64]. Time-resolved sampling, spatially resolved analyses, and predictive modeling approaches will be particularly important for resolving the complex and dynamic nature of ANE-mediated responses. Such approaches will be essential for distinguishing direct signaling events from downstream physiological consequences and for developing more robust mechanistic models of ANE activity.

### 7.7. Challenges in Dose Optimization and Application Strategies

Determining the optimal application rates and timing for ANE remains challenging because plant responses are nonlinear, context-dependent, and highly influenced by signaling dynamics. ANE primarily functions as a regulatory modulator rather than a conventional nutrient input [9,128]. Unlike mineral fertilizers, which often exhibit relatively predictable dose–response relationships, ANE commonly displays hormetic behavior, whereby low to moderate concentrations stimulate plant performance, whereas higher concentrations result in response saturation or reduced efficacy [94,99].

Reported dose–response relationships vary substantially among crops, formulations, and experimental systems, indicating that optimal application rates are highly dependent on genotype, developmental stage, physiological status, and environmental conditions [129,130]. These observations are consistent with the involvement of hormone-associated, redox-regulated, and stress-responsive signaling networks that operate within defined biological thresholds. However, the molecular determinants underlying these thresholds remain poorly understood.

Application strategy further influences ANE efficacy because tissue exposure, uptake dynamics, developmental stage, and environmental conditions affect biological responses. Consequently, nominal concentration alone does not adequately reflect effective biological exposure. Differences among foliar, soil, hydroponic, and seed-treatment approaches further highlight the context-dependent nature of ANE-mediated responses [83,131]. These factors complicate the interpretation of dose–response relationships and hinder comparison among studies employing different application strategies.

Because ANE performance is influenced by dose, formulation, genotype, developmental stage, application strategy, and environmental conditions, universal dosage recommendations are difficult to establish [132,133]. The absence of standardized formulations and mechanistically validated biomarkers further limits the prediction of optimal application regimes. Future studies should integrate physiological, biochemical, transcriptomic, and signaling-related endpoints to establish mechanistically informed dose–response relationships. Greater emphasis on formulation-specific activity, temporal signaling dynamics, and environmental metadata will be required to support predictive modeling and improve reproducibility across production systems. Such approaches will be important for linking biological activity thresholds with molecular responses and improving translation of mechanistic findings into practical application strategies.

### 7.8. Regulatory and Commercial Constraints

The commercial development and regulatory acceptance of ANE-based biostimulants are strongly influenced by challenges associated with product standardization, compositional characterization, and performance validation [134]. Current regulatory frameworks generally emphasize demonstrated biological performance rather than detailed molecular characterization, allowing products with different compositions to be marketed under similar functional claims [135,136,137].

Variability in extraction methodology, biomass source, seasonal conditions, and processing procedures can generate substantial differences in the abundance and structural characteristics of bioactive constituents [12,48,132,138]. Consequently, products derived from the same seaweed species may differ in composition, biological activity, and proposed mechanisms of action, limiting reproducibility and cross-study comparability [139].

A major limitation is the absence of harmonized standards for chemical characterization, biological validation, and performance benchmarking. This constrains comparison among formulations and complicates efforts to establish robust structure–function relationships. Without standardized compositional benchmarks, it remains difficult to determine whether differences in biological responses arise from formulation-specific chemistry or from environmental and experimental factors [140,141].

These limitations are particularly relevant for mechanistic research because compositional variability and inconsistent characterization hinder direct linkage of specific ANE constituents to molecular responses and agronomic outcomes [142,143,144]. Consequently, interpretation of receptor-mediated perception, signaling-associated responses, transcriptional regulation, and downstream physiological effects remains challenging across different ANE formulations.

Thus, the commercialization of ANE-based biostimulants is constrained by two interrelated factors:

1. Substantial compositional variability among formulations, and
2. Regulatory pathways that emphasize functional claims over detailed molecular characterization.

Therefore, improved analytical characterization, standardized production protocols, and stronger integration of molecular, compositional, and agronomic datasets will be essential to support reproducible, evidence-based, and regulatory-compliant development of ANE-based biostimulants. These advances will also facilitate the development of more robust mechanistic models linking ANE composition with biological activity and field performance [145,146].

The major constraints limiting the functional resolution, standardization, and translational deployment of ANE, along with priority research directions, are summarized in Table 5.

## 8. Conclusions

ANE represents a chemically and biologically complex biostimulant system that influences plant growth, productivity, and stress tolerance mainly through modulation of endogenous signaling and regulatory networks rather than direct nutrient supply. Available evidence suggests that ANE-associated responses involve interactions among signaling pathways, hormonal networks, transcriptional regulation, nutrient-acquisition processes, and rhizosphere-mediated effects across multiple biological scales.

Current knowledge supports a proposed model in which ANE functions as a signaling modulator that coordinates molecular, physiological, and ecological responses. However, many components of this framework remain incompletely resolved. In particular, receptor-mediated perception mechanisms, signaling hierarchies linking Ca^2+^-, ROS-, and MAPK-associated pathways, and causal relationships between specific ANE constituents and downstream biological responses remain poorly understood.

The biological activity of ANE appears to arise from interactions among multiple bioactive constituents rather than the independent action of individual compounds. While this complexity may contribute to broad-spectrum activity, it also complicates mechanistic interpretation, structure–function analysis, formulation standardization, and prediction of biological responses. Furthermore, much of the available mechanistic evidence originates from studies using purified ANE-derived polysaccharides or related elicitor systems, highlighting the need for caution when extrapolating proposed mechanisms across chemically diverse ANE formulations. Consequently, several mechanistic relationships discussed in this review should be regarded as working hypotheses requiring further experimental validation.

Future progress will require receptor identification, bioassay-guided fractionation, functional validation, integrated multi-omics approaches, and improved standardization of extract characterization and experimental reporting. Linking molecular responses with physiological performance and field-scale outcomes through coordinated laboratory and field investigations will be essential for establishing robust mechanistic models and supporting the development of reproducible, evidence-based ANE biostimulants for sustainable agriculture. Such advances will help bridge current gaps between extract composition, molecular activity, and agronomic performance.

## Figures and Tables

**Table 1:** Major bioactive constituents of ANE and their principal functional roles in plant regulation and stress response.

Class	Major Constituents	Principal Functional Roles *	References
Structural polysaccharides	Alginates	Water retention, cation binding, rhizosphere conditioning, nutrient retention, generation of bioactive oligosaccharides associated with elicitor activity	[14,15,43]
Storage polysaccharides	Laminarin	Defense priming, stress-associated signaling responses, ROS production, immune responses, stress adaptation	[17,26,44]
Sulfated polysaccharides	Fucoidan	Antioxidant activity, redox balance, defense responses, stress adaptation	[45,46,47]
Osmolytes	Mannitol	Osmotic adjustment, ROS buffering, drought and salinity tolerance	[14,36,39]
Phenolic compounds	Phlorotannins	Antioxidant defense, redox homeostasis, ROS scavenging	[40,41,48]
Pigments (carotenoids)	Fucoxanthin and related carotenoids	Photoprotection, membrane stabilization, antioxidant activity	[13,15]
Phytohormone-like compounds	Auxin-, cytokinin-, gibberellin-, and salicylate-related compounds	Hormonal regulation, root development, growth responses, stress resilience	[9,42,49]
Mineral fraction	K, Ca, Mg, Na, P, Fe, Zn, Mn	Contribution to ionic balance and metabolic function; not considered the primary basis of ANE biostimulant activity	[10,14,43]
Proteins and amino acids	Soluble proteins, peptides, free amino acids	Metabolic constituents and potential signaling-related precursors involved in cellular metabolism	[10,43]
Lipid fraction	Fatty acids and membrane lipids	Membrane integrity, signaling precursors, stress tolerance	[10,43]

* The functional roles listed represent reported or proposed biological activities associated with ANE constituents. Because ANE is a chemically complex mixture, the observed plant responses are generally attributed to interactions among multiple constituents rather than the action of individual compounds in isolation.

**Table 2:** Major extraction approaches used for ANE production and their implications for the extract composition and biostimulant activity.

Extraction Method	Major Recovered Fractions	Structural/Functional Effects	Potential Mechanistic Implications	References
Alkaline extraction	Alginates; soluble polysaccharides	Partial depolymerization under high pH; enrichment of structural polysaccharides	May alter molecular weight distribution and availability of signaling-associated polysaccharide fractions	[43,47,56]
Acid extraction	Fucoidan-rich and laminarin-related fractions	Alters the sulfation pattern and polymer size	May influence elicitor-associated activity through modification of structural features linked to biological responses	[45,46,47]
Hot-water/aqueous extraction	Water-soluble polysaccharides, phenolics, minerals	Produces chemically heterogeneous crude extracts	Generates complex formulations with multiple potential signaling and regulatory constituents	[9,43,56]
Cold-water extraction	Low molecular weight metabolites; phenolics	Preserves heat-sensitive compounds	Preserves metabolites potentially associated with redox regulation and stress-responsive pathways	[47,56]
Enzyme-assisted extraction	Polysaccharides, phenolics, intracellular metabolites	Preserves structural integrity under mild conditions	May improve preservation of bioactive structures associated with signaling responses	[47,53,57]
Ultrasound-assisted extraction	Polysaccharides, phenolics, soluble metabolites	Enhances cell disruption and mass transfer	May alter the relative abundance of signaling-associated metabolites through enhanced extraction efficiency	[30,44]
Microwave-assisted extraction	Phenolics, pigments, soluble carbohydrates	Rapid thermal disruption of biomass	May modify the composition of recovered metabolites and influence biological activity	[30,50]
Supercritical fluid extraction	Lipids, pigments, antioxidants	Selective recovery of nonpolar metabolites	Facilitates enrichment of antioxidant- and lipid-associated constituents with potential regulatory functions	[30,50]
Sequential/biorefinery extraction	Alginates, fucoidan, phenolics, minerals	Recovery of multiple functional components	Supports development of chemically characterized formulations for structure–function studies	[14,55]

**Table 3:** Principal molecular and regulatory processes associated with ANE-mediated responses, representative markers, biological relevance, and the primary sources of mechanistic evidence.

Regulatory Process	Representative Genes/Pathways	Major ANE-Induced Responses	Biological Relevance	Evidence Type *	References
Early elicitor signaling	Cytosolic Ca^2+^ flux, oxidative burst, extracellular alkalinization, MAPK activation	Association with early signaling responses including Ca^2+^ flux, ROS generation, and MAPK activation	Signal amplification and defense initiation	Primarily derived from purified ANE-derived polysaccharides (e.g., laminarin) with limited supporting evidence from whole ANE studies	[17,26]
MAPK signaling	MAPK-associated kinase pathways	Modulation of MAPK-associated stress and defense signaling	Integration of extracellular signals with transcriptional regulation	Transcriptomic, signaling, and purified polysaccharide studies	[28,92]
Hormonal signaling	Auxin, cytokinin, JA, SA, ET, ABA pathways	Modulation of hormone-responsive pathways	Regulation of growth, development, and tolerance	Transcriptomic and gene-expression studies	[28,29,49]
Auxin-responsive development	AUX/IAA, ARF-related pathways	Increased expression of auxin-responsive genes	Root development and developmental plasticity	Gene-expression and developmental studies	[29]
Nutrient transport	NRT1.1, NRT2.1, BnSultr4.1	Increased expression of nutrient transporter genes	Improved nutrient uptake and use efficiency	Whole ANE studies with gene-expression support	[20,84]
Nitrogen assimilation	NR, NiR, GS, GOGAT	Association with nitrogen assimilation-related pathways	Improved nitrogen metabolism and nutrient-use efficiency	Whole ANE studies, biochemical assays, and gene-expression analyses	[84]
Carbon metabolism and photosynthesis	Photosynthesis-associated genes, Calvin cycle pathways	Association with changes in carbon metabolism and photosynthetic processes	Biomass accumulation and growth enhancement	Transcriptomic and physiological studies	[28]
Redox and antioxidant regulation	SOD, CAT, POD, APX, glutathione pathway	Association with antioxidant responses and redox regulation	ROS detoxification and stress tolerance	Biochemical, physiological, and oxidative stress studies	[28,93]
Phenylpropanoid and secondary metabolism	PAL, phenylpropanoid biosynthesis genes	Increased expression of phenylpropanoid-associated pathways and secondary metabolism	Antioxidant accumulation and defense reinforcement	Transcriptomic, metabolomic, and physiological studies	[28,77]
Pathogenesis-related defense	PR proteins, chitinase, β-1,3-glucanase, ISR/SAR markers	Association with defense-related gene expression and enzyme activity	Immune priming and pathogen resistance	Whole ANE studies and purified polysaccharide studies; mechanistic pathways incompletely resolved	[17,23,73]
Global transcriptional regulation	Differentially expressed genes (DEGs)	Differential gene expression and transcriptional responses	Integration of growth- and stress-related gene networks	Transcriptomic studies (RNA-seq and DEG analyses)	[28,64,70]

Abbreviations: ABA, abscisic acid; ANE, *Ascophyllum nodosum* extract; APX, ascorbate peroxidase; CAT, catalase; GS, glutamine synthetase; GOGAT, glutamate synthase; ISR, induced systemic resistance; MAPK, mitogen-activated protein kinase; NiR, nitrite reductase; NR, nitrate reductase; POD, peroxidase; PR, pathogenesis-related; ROS, reactive oxygen species; SAR, systemic acquired resistance; SOD, superoxide dismutase; SA, salicylic acid; JA, jasmonic acid; ET, ethylene. * Evidence type indicates the primary source of mechanistic support. Several signaling mechanisms remain inferred from studies using purified ANE-derived polysaccharides (e.g., laminarin and fucoidan) or related elicitor systems and should not be interpreted as universally applicable to all ANE formulations.

**Table 4:** Representative agronomic responses associated with ANE application across different crop systems and their proposed links to underlying molecular, physiological, and metabolic processes.

Crop System	Application Strategy	Major Agronomic Responses	Functional Relevance	References
Tomato (*Solanum lycopersicum*)	Foliar spray; soil drench	Increased plant growth, biomass accumulation, fruit yield, and drought tolerance	Improved growth regulation and stress tolerance	[20,21]
Lettuce (*Lactuca sativa*)	Hydroponic supplementation	Increased leaf area and fresh biomass	Enhanced vegetative growth and nutrient-use efficiency	[95]
Maize (*Zea mays*)	Root application	Enhanced root growth and nutrient uptake under phosphorus limitation	Improved nutrient acquisition and root plasticity	[22]
Arabidopsis (*Arabidopsis thaliana*)	Media supplementation	Increased root branching and stress-responsive molecular regulation	Model system validation of ANE-mediated signaling responses	[81,99]
Wheat and cereals	Foliar application	Improved nutrient-use efficiency and maintenance of yield under reduced nitrogen input	Enhanced nutrient metabolism and agronomic sustainability	[85]
Grapevine (*Vitis vinifera*)	Foliar application	Improved photosynthesis, yield, and stress tolerance	Enhanced physiological performance under field conditions	[97,100]
Strawberry (*Fragaria × ananassa*)	Foliar application	Increased phenolic and flavonoid accumulation	Improved fruit quality and antioxidant metabolism	[74]
Okra (*Abelmoschus esculentus*)	Foliar application	Enhanced chlorophyll content, antioxidant activity, and drought tolerance	Improved abiotic stress resilience	[28]
Avocado (*Persea americana* cv. Hass)	Foliar application	Increased biomass accumulation, gas exchange, and yield	Improved physiological performance and productivity	[101]

**Table 5:** Major limitations constraining the functional interpretation and translational application of ANE, together with priority research directions.

Major Challenge	Key Limitation	Priority Research Needs	References
Extract variability and lack of standardization	Compositional heterogeneity associated with biomass source, seasonal variation, extraction methodology, and formulation characteristics	Batch fingerprinting, compositional standards, reference materials, and activity-based validation assays	[43,46,47]
Unresolved perception mechanisms	Lack of identified receptors and ligand-specific recognition pathways for ANE-derived bioactive compounds	Receptor identification, ligand-binding studies, mutant analysis, live-cell imaging, and structure–activity investigations	[26,105]
Incomplete signaling and transcriptional networks	Limited understanding of interactions among Ca^2+^, ROS, MAPK-associated pathways, hormonal regulation, and transcriptional responses	Time-resolved phosphoproteomics, biosensors, network analysis, functional genomics, and genetic validation	[28,92]
Multi-component interaction complexity	Difficulty distinguishing synergistic, additive, and antagonistic effects among ANE constituents	Bioassay-guided fractionation, factorial mixture studies, machine learning-assisted interaction analysis, and causal modeling	[49,110]
Limited multi-omics integration	Insufficient integration of transcriptomics, proteomics, metabolomics, phosphoproteomics, ionomics, and microbiome analyses	Systems-biology approaches and integrated multi-omics platforms for identification of regulatory hubs and biomarkers	[28,123]
Experimental inconsistency and dose dependency	Variation in dose, application strategy, environmental conditions, and extract characterization limits reproducibility	Harmonized reporting frameworks, dose normalization, standardized protocols, and mechanistically informed dose–response models	[56,94,99]
Field validation and environmental complexity	Controlled-environment responses do not always predict field performance because of G × E × M interactions and microbiome effects	Multilocation field trials integrating molecular, microbiome, environmental, and agronomic datasets	[43,99,122]
Standardization-to-deployment gap	Limited linkage among molecular characterization, mechanistic validation, product standardization, and field performance	Integrated compositional, molecular, and agronomic validation frameworks supporting evidence-based product development	[94,134,141]

## Data Availability

No new data were created or analyzed in this study. Data sharing is not applicable to this article.
